# Full-length haplotype reconstruction of *CD36* by long-read sequencing: uncovers a novel structural variant

**DOI:** 10.1186/s12864-026-12701-2

**Published:** 2026-02-27

**Authors:** Shuang Liang, Tong Liu, Huatao Che, Wenxia Xia, Weiyi Fu, Fan Wu, Liyan Sun, Yongshui Fu, Faming Zhu, Dawei Cui

**Affiliations:** 1 Institute of Transfusion Medicine, Shenzhen Blood Center, Shenzhen, China; 2 DAFEI biotechnology Co., Ltd, Guangzhou, China; 3 Guangzhou First People’s Hospital, Guangzhou, China; 4 Blood Transfusion Medicine Research Institute, Blood Center of Zhejiang Province, Hangzhou, China; 5 Department of Blood Transfusion, The First Affiliated Hospital, Zhejiang University School of Medicine, Hangzhou, China

**Keywords:** CD36, Full-length haplotype, Long-read sequencing, Structural variant

## Abstract

**Purpose:**

CD36 deficiency predisposes to fetal/neonatal alloimmune thrombocytopenia (FNAIT) and platelet transfusion refractoriness (PTR), yet its genetic architecture remains incompletely understood. In particular, the contribution of large structural variants has been difficult to assess using conventional genotyping approaches. This study aimed to reconstruct full-length *CD36* haplotypes using long-read sequencing and to systematically characterize its genetic architecture.

**Methods:**

We developed a long-read sequencing approach to reconstruct full-length (~ 77 kb) *CD36* haplotypes from four overlapping amplicons. Samples from 43 CD36-deficient individuals (14 type I, 29 type II) were analyzed. Structural variants were validated via PacBio whole-genome sequencing, platelet CD36 expression was quantified by flow cytometry, and a gap-PCR assay screened 600 blood donors for large deletions.

**Results:**

Full-length *CD36* haplotypes were reconstructed for the first time using overlapping amplicons. Candidate pathogenic variants were identified in all 28 haplotypes from type I deficiency samples, including a novel structural variant (c.1-15966_c.120 + 3887 delinsCCAATGCTAAGGTTGA, 19,971 bp deletion-insertion) spanning intron 1–3 that eliminates exons 2/3 and the canonical translation initiation site. Among 58 haplotypes from type II cases, potentially pathogenic variants were identified in only 26/58 (44.8%) (including one haplotype carrying the novel structural variant), while 32/58 (55.2%) lacked detectable variations. Gap-PCR screening revealed a 0.50% carrier frequency for this structural variant in blood donors. All heterozygous carriers showed normal platelet CD36 expression, indicating absence of haploinsufficiency.

**Conclusions:**

This study establishes a robust PolySeq nanopore sequencing-based framework for full-length *CD36* haplotype reconstruction and identifies a large structural deletion as a major, previously underrecognized candidate genetic mechanism underlying type I CD36 deficiency. Our findings suggest that many unresolved cases of type I deficiency may be attributable to cryptic structural variants missed by conventional methods, highlighting the necessity of long-read sequencing for accurate molecular diagnosis.

**Supplementary Information:**

The online version contains supplementary material available at 10.1186/s12864-026-12701-2.

## Key Points

• A long-read sequencing approach was established and successfully and firstly reconstructed the complete ~ 77kb *CD36* haplotype, enabling comprehensive detection of structural and phased variants that are inaccessible to conventional genotyping.

• A novel 19,971 bp deletion removing exons 2–3 and the canonical translation initiation site represents a major genetic mechanism underlying type I *CD36* deficiency and is frequently missed by short-range PCR and Sanger sequencing.

**Supplementary Information:**

The online version contains supplementary material available at 10.1186/s12864-026-12701-2.

## Introduction

CD36, also known as glycoprotein IV (GPIV), is an 88-kDa transmembrane protein expressed on a wide range of cell types including platelets, monocytes, endothelial cells, and erythroid precursors, though expression on mature red blood cells (RBCs) is relatively low [1, 2]. Initially identified as a platelet membrane component, CD36 has since been implicated in diverse physiological functions including lipid metabolism, innate immunity, and cell adhesion, and it also serves as a key antigen target in alloimmune thrombocytopenia disorders [3–5].

CD36 deficiency is classified into two phenotypes: type I, characterized by complete absence of expression on both platelets and monocytes, and type II, defined by selective absence on platelets [6, 7]. Although individuals with CD36 deficiency are often clinically asymptomatic, exposure to CD36-positive cells through blood transfusion or pregnancy can lead to alloimmunization and the production of anti-CD36 antibodies [8, 9]. These antibodies are known to cause serious immune-mediated complications such as FNAIT, PTR, and posttransfusion purpura [8, 10–12]. Importantly, CD36-negative phenotypes vary in prevalence across populations, reaching up to 11% in certain Asian cohorts, and have become increasingly relevant to transfusion practice in regions with active donor surveillance [6, 13].

The *CD36* gene, located on chromosome 7q21.11, spans approximately 77 kb and includes 15 exons, 12 of which are coding [14]. Although over 30,000 variants are listed in the dbSNP database, only a small subset is known to affect protein expression [6]. Most studies have focused on coding variants and selected single nucleotide variants (SNVs), while the broader haplotype structure and upstream regulatory regions remain understudied [13, 15, 16]. Using PacBio sequencing, Xia et al. obtained ~ 40 kb haplotypes from exon 2 to the 3’ untranslated region (UTR) [17]. However, in some type I deficiency cases, causative variants could not be identified within these regions, suggesting that critical regulatory elements might reside in intron 1 or adjacent noncoding regions [18, 19].

In this study, we used four overlapping long-range polymerase chain reaction (PCR) amplicons (20 ~ 23 kb each) to generate full-length (~ 77 kb) haplotype sequences of the *CD36* gene using the Polyseq nanopore sequencing platform [20]. Applying this approach, we analyzed samples from 14 individuals with type I CD36 deficiency and 29 with type II deficiency. In three type I and one type II CD36 deficiency samples, we detected potential structural variants located in intron 1. Among them, two cases were further validated by PacBio whole-genome sequencing, which confirmed a 19,971 bp deletion (c.1-15966_c.120 + 3887delinsCCAATGCTAAGGTTGA) with precise breakpoint resolution. Finally, we screened 300 routine blood donors using a PCR-based assay and estimated the allele frequency of this large deletion haplotype to be approximately 0.5%.

## Methods and materials

### Sample collection

**Fig. 1:**
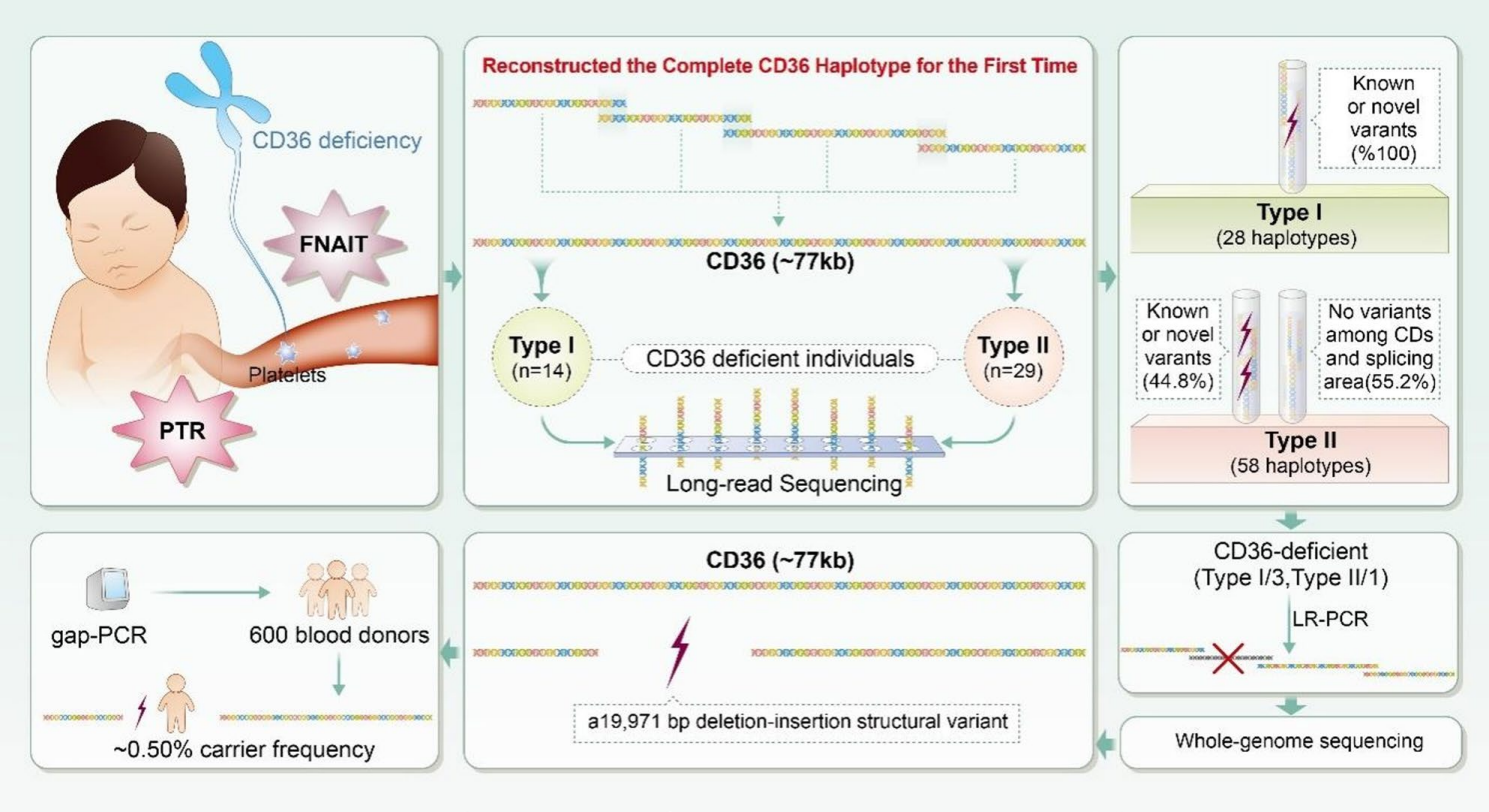
Schematic overview of identification for the full-length *CD36* gene

Fourteen type I and twenty-nine type II CD36-deficient samples were randomly selected from the Shenzhen Blood Center CD36 Deficiency Bank. An additional 600 normal samples were collected from routine healthy donors at the same center. Peripheral blood (5.0 mL) was collected in ethylenediaminetetraacetic acid (EDTA) anticoagulant tubes for genomic DNA extraction and flow cytometry analysis. A graphical overview of this study is provided in Fig. 1. All donors were of Han ethnicity and all samples were obtained with informed consent under ethical approval from the Shenzhen Blood Center Ethics Committee (SZBCMEC-2024-047).

### Genomic DNA extraction

Genomic deoxyribonucleicacid (DNA) was isolated from EDTA-anticoagulated peripheral blood using the MagNA Pure LC DNA Isolation Kit I (Roche Diagnostics). DNA purity (A₂₆₀/A₂₈₀ ratio 1.8-2.0) and concentration were assessed by spectrophotometry (NanoDrop 8000), and extracts were normalized to 30 ng/µL in Tris-HCl/EDTA buffer for downstream use.

### LR-PCR for full-length *CD36* gene

**Fig. 2:**
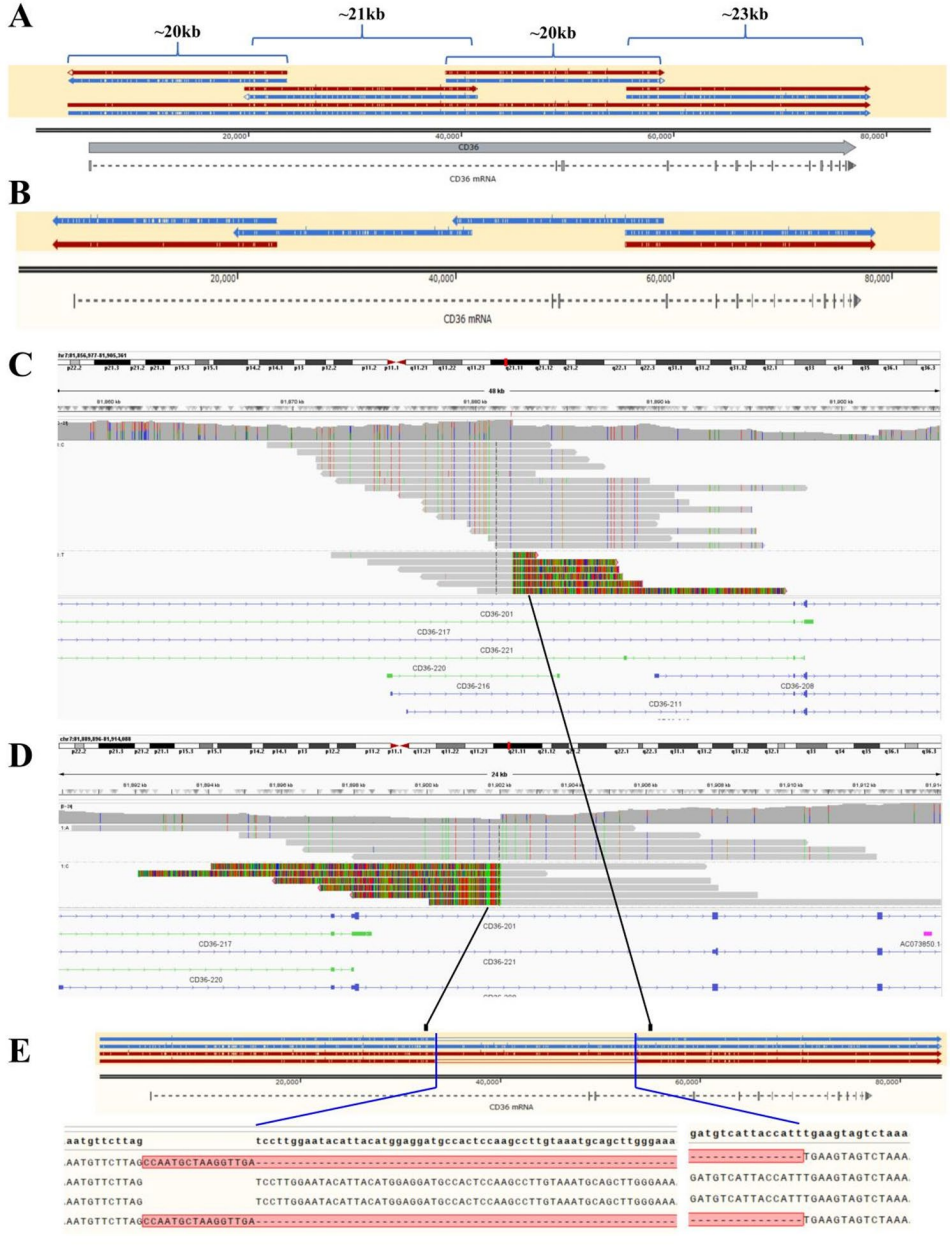
Schematic of the structural variant in the *CD36* gene. **A** Targeted amplification and sequencing of the full-length *CD36* gene. Red and blue arrows depict alignment of haplotype sequences to the *CD36* reference (T2T-CHM13v2.0GRCh38). White bars within arrows indicate base differences or insertions relative to the reference; SNP differences within overlapping amplicon regions enabled phasing of the four fragments into complete haplotypes. **B** Four deficient samples showed a consistent pattern: one haplotype amplified all four amplicons, while the other failed to amplify amplicons 2 and 3. A representative sample is shown. Red and blue arrows represent alignment to the *CD36* reference sequence (T2T-CHM13v2.0). Differences from the reference are indicated by white bars within arrows; blue/red lines/triangles denote insertions. **C**-**D** IGV visualization of PacBio whole-genome sequencing (WGS) data at the breakpoint regions. To reduce the impact of random sequencing errors, the Hide small indels parameter was set to 5 bp. Reads were grouped by nearby heterozygous SNPs to enable haplotype resolution at the two breakpoints. **C** shows the right breakpoint region (grouped by base at chr7:81,881,952), and (**D**) shows the left breakpoint region (grouped by base at chr7:81,902,087). This approach clearly distinguishes the haplotype carrying the large deletion from the wild-type haplotype, allowing direct visualization of the structural variant in the heterozygous state. **E** Assembled PacBio sequences from two samples. The blank region represents the deleted segment. Nucleotide sequences at the precise breakpoints are shown

Primer sets (*CD36* 1 Forward/Reverse-4 Forward/Reverse) were designed to amplify regions spanning from the 5’ UTR to the 3’ UTR of the *CD36* gene, covering a total length of approximately 77 kb and including selected intronic sequences. Four sets were developed to generate amplicons ranging from 21 to 23 kb in length, with ≥ 3 kb overlap between adjacent fragments (Fig. 2A). All primer sequences are listed in Table 1. Long-range PCR (LR-PCR) was performed using ApexHF HS DNA Polymerase CL (Accurate Biology, AG12204) according to the manufacturer’s instructions. PCR cycling consisted of a three-step program: 98 °C for 10 s, 65 °C for 10 s, and 72 °C for 3.5 min, for 30 cycles.

**Table 1:** Primer pairs used for full-length *CD36* gene amplification and gap-PCR assay

Primer	Sequence (5’→3’)	Size	Coverage	Usage
CD36 1F	ctccctcaccacctatccctataagct	20.6 kb	5’UTR~exon 1	LR-PCR primers for LRS
CD36 1R	ctgccactgcttcatcaaccaacttta			
CD36 2F	caaagtggctacggcgattgtaaggatc	22 kb	intron 1	
CD36 2R	acagtagtgtcacctcccgtcatctgg			
CD36 3F	tccaaattcagactcggattccattcat	20.6 kb	exon 2 ~ 3	
CD36 3R	accttaccatacacttgcagcagaaat			
CD36 4F	ggcttagacagtaaatgctatgaaccaag	23 kb	exon 4 ~ 14	
CD36 4R	caccgtcttacactttgctacttcttaca			
CD36 5F	GTGTCAAACTGTCATCGGGGTG	20 kb	exon 2 ~ 3	deletion-flanking primers for gap-PCR
CD36 5R	GACCCTTTTCCACAGTGCTTTG			
CD36 6F	CAGAGAGATGTGGAGGGGAGTT	290 bp	intron 7	control primers for gap-PCR
CD36 6R	TCTTGCCCCTTCCTCCCTTTA			

*F* Forward, *R* Reverse

### Library preparation, nanopore sequencing and bioinformatic analysis

Library preparation was performed using the commercial PY-DTB101/102 and PY-BLP101 kits (Polyseq Biotech Co. Ltd., China) according to the manufacturer’s instructions. Prepared libraries were loaded onto PY-NFC001 flow cells and sequenced for 12 h on the PolyseqOne nanopore sequencing platform (www.polyseq.com). Basecalling and demultiplexing were performed using Kant v1.0.1 in high-accuracy mode (processing rate: 420 bases per second). Raw reads underwent demultiplexing with automated barcode identification. For *CD36* sequence analysis, reads were first aligned to the T2T-CHM13v2.0 reference genome and error-corrected using NanoFix-AI (DAFEI Biotechnology, Guangzhou, China) [21]. FASTQ reads corresponding to individual *CD36* alleles were extracted using bedtools (v2.30.0). A custom Python workflow was then employed to convert these reads into clustered BAM files. Finally, the clustered reads were re-aligned to the *CD36* reference gene sequence using pbmm2.

### Whole-genome sequencing analysis

Long-read whole-genome sequencing was performed on the PacBio Revio platform (Pacific Biosciences), generating 31.2 Gb and 52.4 Gb of HiFi reads with median qualities of Q35. Coverage depth across the *CD36* gene was 25× and 41×, respectively. Reads were aligned to the human reference genome (CHM13v2.0) using minimap2 (2.28-r1209, https://github.com/lh3/minimap2) under default parameters. Subsequently, reads spanning the *CD36* region were extracted using bedtools (v2.31.1; https://github.com/arq5x/bedtools2). De novo assembly was then performed with hifiasm (v0.24.0-r702; https://github.com/chhylp123/hifiasm) using the following parameters: “-n 2 -t 96 --n-hap 2 -s 0.75 -z 50 -u 1”. Large deletions were detected using software developed by DAFEI Biotechnology (Guangzhou, China). Coverage was assessed with mosdepth, and deletion breakpoints/junctions were manually verified using the Integrative Genomics Viewer (IGV). *CD36* sequence data were visualized in SnapGene for clear presentation of structural findings.

### Population screening for 19,971-bp deletion-insertion structural variant

We developed a multiplex gap-PCR assay to screen 600 blood donors for the recurrent 19,971 bp deletion-insertion structural variant spanning exons 2–3. Two primer pairs were designed based on the *CD36* gene sequence (GenBank ID: NG_008192.1): control primers (*CD36* 6 F/R) amplifying a 333-bp fragment in intron 7, and deletion-flanking primers (*CD36* 5 F/R) positioned 571 bp upstream (5 F) and 261 bp downstream (5R) of the deletion boundaries. All primer sequences are listed in Table 1. PCR amplification was performed in multiplex format under the following conditions: 30 cycles of 98 °C for 10 s, 63 °C for 30 s, and 72 °C for 1 min, with pre-validated positive and negative controls included in each run. All candidate positives underwent confirmation through nanopore sequencing (Polyseq platform) to verify breakpoint identity with whole-genome sequencing data and determine zygosity status.

### Flow cytometric analysis for CD36 expression on platelets

CD36 surface expression on platelets was assessed by flow cytometry using fresh whole blood samples processed within 24 h of collection. Platelet-rich plasma (PRP) was isolated from EDTA-anticoagulated blood by centrifugation at 200 ×g for 10 min at room temperature. Following three washes with phosphate buffered saline (PBS) containing 0.5% bovine serum albumin (BSA) and 1 mM EDTA (PBS-BSA-EDTA), platelets were resuspended at 1 × 10⁷ cells/ml. For staining, 20 µL platelet suspension was incubated with 2 µL FITC-conjugated anti-CD36 antibody (clone FA6-152; BD Biosciences, USA) for 20 min at room temperature in the dark. After three washes with PBS-BSA-EDTA, cells were resuspended in 500 µL PBS. Analysis was performed on a FACSCanto II flow cytometer (BD Biosciences) with platelets gated by characteristic forward/side scatter (FSC/SSC) profiles. CD36 expression was quantified as mean fluorescence intensity (MFI) using FACSDiva software v8.0.1 (BD Biosciences).

## Results

### Reconstruction of full-length *CD36* haplotypes by nanopore sequencing

Full-length (~ 77 kb) *CD36* haplotypes were reconstructed by SNP-based phasing of four overlapping 21–23 kb amplicons, sequenced using the Polyseq nanopore platform (Fig. 2A). Using this approach, we analyzed samples from 14 type I and 29 type II CD36-deficient individuals. All identified exonic variants (including those within 10 bp of exon-intron boundaries) present on each haplotype are summarized in Table 2. This represents the first successful reconstruction of the complete ~ 77 kb *CD36* haplotype, providing a comprehensive view of its genetic architecture.

**Table 2:** Genetic variations in *CD36* exons among haplotype sequences from CD36 deficient samples

Sample ID	Haplotype 1	Haplotype 2	Type
I-01	c.-132 A > C	c.430-1G > C; c.-132 A > C	I
I-02	c.-132 A > C	c.1228_1239delATTGTGCCTATT	I
I-03	c.-132 A > C	c.1229T > C*; c.-132 A > C	I
I-04	c.332_333delCA; c.-132 A > C	c.1228_1239delATTGTGCCTATT	I
I-05	c.332_333delCA; c.-132 A > C	c.1228_1239delATTGTGCCTATT	I
I-06	c.332_333delCA; c.-132 A > C	c.1228_1239delATTGTGCCTATT	I
I-07	c.380 C > T	c.1228_1239delATTGTGCCTATT	I
I-08	c.430–2 A > G	c.609 + 7 A > G*; c.-132 A > C	I
I-09	c.1228_1239delATTGTGCCTATT	c.1156 C > T	I
I-10	c.1228_1239delATTGTGCCTATT	c.1156 C > T; c.1409 C > T	I
I-11	c.1228_1239delATTGTGCCTATT	c.1228_1239delATTGTGCCTATT	I
I-12	c.1-15966_c.120 + 3887 delinsCCAATGCTAAGGTTGA	c.1006 + 2T > G	I
I-13	c.1-15966_c.120 + 3887 delinsCCAATGCTAAGGTTGA	c.1156 C > T; c.1409 C > T	I
I-14	c.1-15966_c.120 + 3887 delinsCCAATGCTAAGGTTGA	c.1163 A > T	I
II-01	c.-132 A > C	-	II
II-02	c.-132 A > C	-	II
II-03	c.220 C > T; c.-132 A > C	c.-132 A > C	II
II-04	c.275 C > T	-	II
II-05	c.332_333delCA	-	II
II-06	c.332_333delCA; c.-132 A > C	-	II
II-07	c.332_333delCA; c.-132 A > C	-	II
II-08	c.332_333delCA; c.-132 A > C	-	II
II-09	c.332_333delCA; c.-132 A > C	-	II
II-10	c.332_333delCA; c.-132 A > C	-	II
II-11	c.429 + 3insG*	-	II
II-12	c.847G > A	-	II
II-13	c.1156 C > T	-	II
II-14	c.1156 C > T	-	II
II-15	c.1156 C > T; c.1409 C > T	-	II
II-16	c.1228_1239delATTGTGCCTATT	-	II
II-17	c.1228_1239delATTGTGCCTATT	-	II
II-18	c.1228_1239delATTGTGCCTATT	c.-132 A > C	II
II-19	c.1228_1239delATTGTGCCTATT	c.609 + 7 A > G*; c.-132 A > C	II
II-20	c.1228_1239delATTGTGCCTATT; c.-132 A > C	-	II
II-21	c.1340_1343dupTCTT	-	II
II-22	c.1-15966_c.120 + 3887 delinsCCAATGCTAAGGTTGA	c.681 C > A*	II
II-23	-	-	II
II-24	-	-	II
II-25	-	-	II
II-26	-	-	II
II-27	-	-	II
II-28	-	-	II
II-29	-	-	II

*Represents the new variants identified in this study

### Variant spectrum and structural variants in type I and II CD36 deficiency

Analysis of the haplotypes derived from 14 type I deficiency samples revealed that all harbored either previously reported potentially pathogenic point variations or complex structural variants [22], including 13 compound heterozygous variants and 1 homozygous variant. The most frequent variant was c.-132 A > C and c.1227delTATTGTGCCTAT, both detected in 32.1% (9/28) of haplotypes. The remaining 35.7% (10/28) of haplotypes harbored a spectrum of other variants, including the novel 19,971-bp deletion (10.7%, 3/28) and multiple other point variations and indels as detailed in Table 2. Importantly, this deletion could not be detected by our conventional Sanger sequencing previously (data not shown), as routine exon-based assays amplify intact exons from the wild-type allele, thereby masking large heterozygous deletions.

In contrast, among the 28 type II deficiency samples, 22 exhibited variants in the *CD36* gene (18 compound heterozygous variants and 4 heterozygous variants), the predominant variant was c.-132 A > C, identified in 20.7% (12/58; assuming a comparable denominator context, or specify total type II haplotypes if known). Thus, we successfully obtained complete haplotype sequences in 43 CD36 deficiency samples using the Polyseq nanopore platform, enabling phased variant calling that was not achievable by previous methods. All haplotype sequences have been submitted to the NCBI database (Table 3).

**Table 3:** Genetic variations of the *CD36* gene in CD36 deficient samples

Genetic variations	Exon/Intron	Type I deficiency (*n* = 28)		Type II deficiency (*n* = 58)		Accession no.
		*n*	Frequency	*n*	Frequency	
c.220 C > T	exon 4	0	0.00%	1	1.67%	PV866985
c.275 C > T	exon 4	0	0.00%	1	1.67%	PV866988
c.332_333delCA	exon 5	3	10.00%	6	10.00%	PV808479
c.380 C > T	exon 5	1	3.33%	0	0.00%	PV808480
c.429 + 3insG	intron5	0	0.00%	1	1.67%	PV866987
c.430-1G > C	intron5	1	3.33%	0	0.00%	PV866984
c.430–2 A > G	intron5	1	3.33%	0	0.00%	PV832518
c.609 + 7 A > G	intron6	1	3.33%	1	1.67%	PV808482
c.681 C > A	exon 7	0	0.00%	1	1.67%	PV855215
c.847G > A	exon 10	0	0.00%	1	1.67%	PV866986
c.1006 + 2T > G	intron10	1	3.33%	0	0.00%	PV866989
c.1156 C > T	exon 12	1	3.33%	2	3.33%	PV808481
c.1156 C > T; c.1409 C > T	exon 12, 14	2	6.67%	1	1.67%	PV855214
c.1163 A > T	exon 12	1	3.33%	0	0.00%	PV855217
c.1227delTATTGTGCCTAT	exon 13	9	30.00%	5	8.33%	PV786778
c.1229T > C	exon 13	1	3.33%	0	0.00%	PV855218
c.1340_1343dupTCTT	exon 14	0	0.00%	1	1.67%	PV855216
c.1-15966_c.120 + 3887 delinsCCAATGCTAAGGTTGA	exon 2–3	3	10.00%	1	1.67%	PV866990

### A novel structural variant spanning *CD36* exons 2–3

In four CD36-deficient samples (3 type I, 1 type II), nanopore sequencing revealed consistent amplification failure of amplicons 2–3 in one haplotype (Fig. 2B), suggesting structural variants. To further characterize this inspection, we performed whole-genome sequencing on two deficient samples (I-13 and II-22) using the PacBio Revio platform. This generated 31.2 Gb and 52.4 Gb of HiFi reads, respectively, with a median quality score of Q35; the majority of reads exceeded 10 kb in length (Figure S1). Visualization using IGV revealed a sharp drop in coverage and disrupted alignment across the second half of intron 1 (Fig. 2C-D). Subsequent de novo assembly identified a 19,971 bp deletion-insertion structural variant (c.1-15966_c.120 + 3887delinsCCAATGCTAAGGTTGA, NG_008192.1: g.33588_g.53560delinsCCAATGCTAAGGTTGA) in one haplotype of each sample. This deletion spans positions c.1-15966 (following a 16-bp insertion) to c.120 + 3887 (relative to the *CD36* reference sequence, Fig. 2E), thereby removing exons 2 and 3, including the translation initiation site located in exon 3.

### Population screening for 19,971-bp deletion-insertion structural variant

**Fig. 3:**
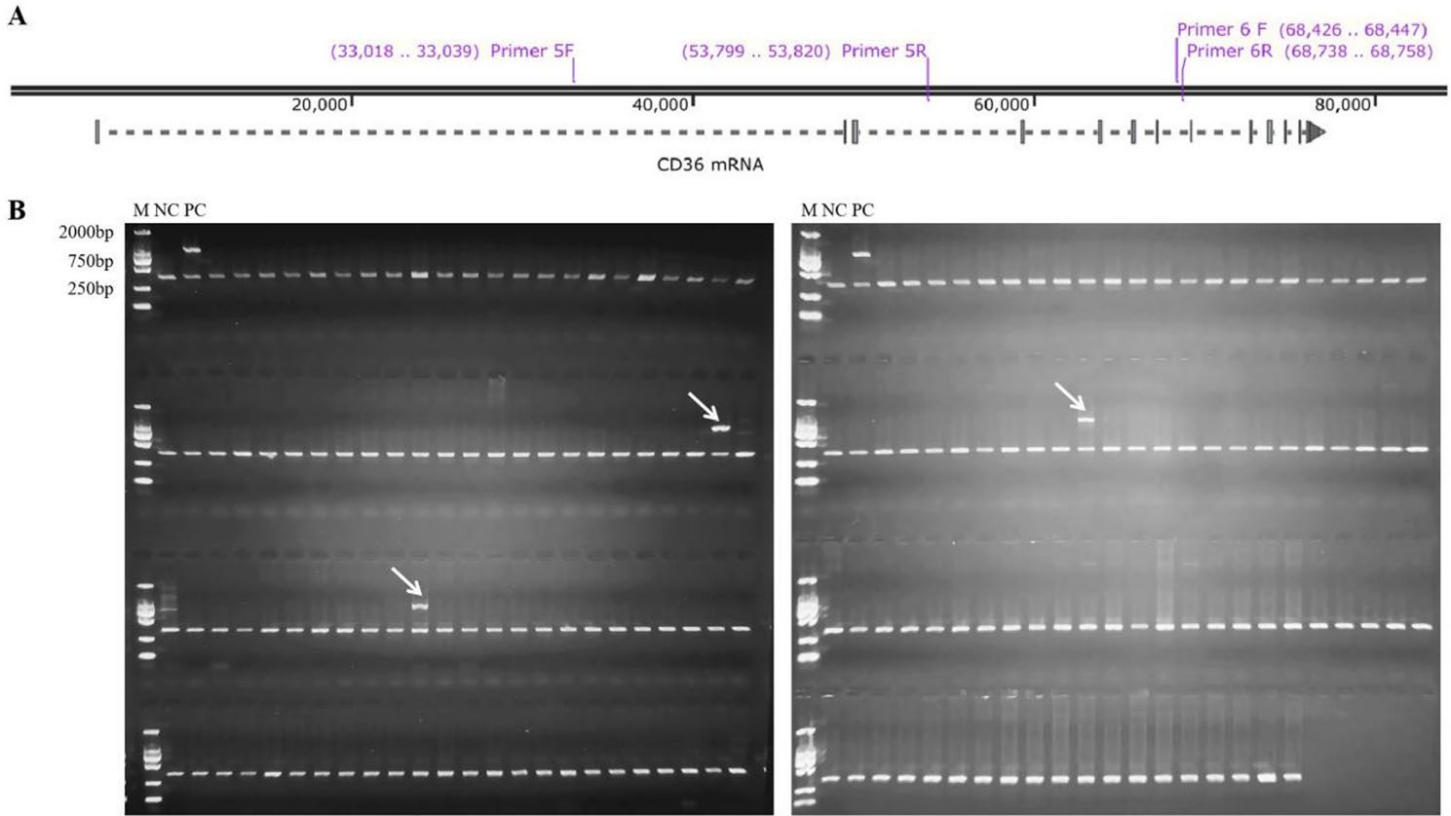
Gap-PCR screening for the structural variant in blood donors. **A** Primer design schematic. CD36 5 F/R primers flank the deletion breakpoints; CD36 6 F/R primers (intron 7) amplify a 333-bp internal control fragment. **B** Agarose gel electrophoresis. Lane M: Marker; NC: Negative control; PC: Positive control; Arrows indicate three heterozygous carriers (Sample 69, 81 and 223) showing both 333-bp control and 832-bp deletion-specific bands

**Fig. 4:**
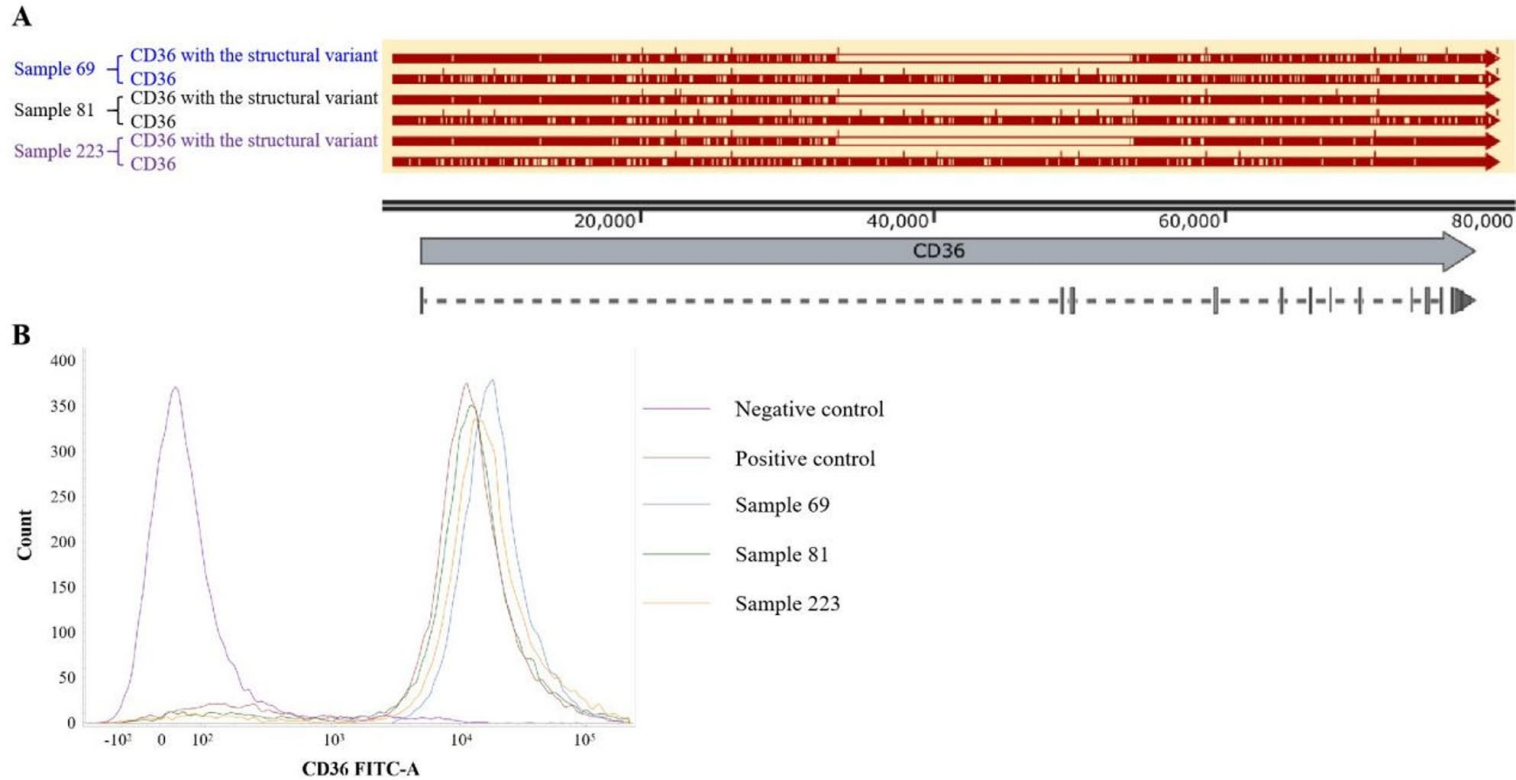
Molecular and phenotypic characterization of heterozygous carriers of the structural variant. **A** Nanopore sequencing confirmation of identical deletion breakpoints in three carriers. Haplotype sequences spanning the deletion junction (dashed box) show concordance with deficient cases. **B** Flow cytometric analysis of platelet CD36 surface expression. Histogram overlay demonstrates preserved expression in heterozygous carriers (blue: Sample 69; green: Sample 81; yellow: Sample 223) compared to positive (red, wild-type) and negative (purple, CD36-deficient) controls

Under the established gap-PCR conditions, amplification of the wild-type allele (20.8-kb) consistently failed, whereas the deletion allele yielded a distinct 832-bp fragment. Consequently, samples exhibiting both the 333-bp control and 832-bp bands were classified as potential carriers (Fig. 3A). Screening of 600 blood donors using this assay identified three heterozygous carriers (Fig. 3B), indicating a population carrier frequency of 0.50% (3/600), and the full uncropped Gels and Blots image(s) were displayed as “Supplementary file”. Nanopore sequencing confirmed that the breakpoints in these carriers were identical to those observed in the CD36-deficient cases (Fig. 4A). Critically, flow cytometric analysis demonstrated preserved CD36 antigen expression on platelets in all three heterozygotes (Fig. 4B), indicating that the deletion alone does not cause haploinsufficiency.

## Discussion

CD36 deficiency poses a significant risk in transfusion medicine due to its potential to induce alloantibodies that can cause PTR and FNAIT, particularly in Asian and African populations where its prevalence is higher [5–7]. Despite its clinical importance, the genetic architecture of CD36 deficiency remains incompletely understood. Although multiple point variations and small indels in exonic and splice-site regions have been reported, these do not fully explain the observed phenotypic variation [8, 10, 11, 13]. Evidence suggests that structural variants and regulatory elements in noncoding regions may contribute to the pathogenesis, especially in type I deficiency [12, 14]. Additionally, the highly polymorphic nature of the *CD36* locus complicates the establishment of clear genotype-phenotype correlations [6].

In this study, we applied long-read sequencing to reconstruct full-length *CD36* haplotypes, achieving comprehensive resolution of the ~ 77 kb locus for the first time. By integrating the previously reported c.-132 A > C promoter variant and c.1228-1239delATTGTGCCTATT deletion variant [13], with a newly identified recurrent 19,971-bp deletion identified here, we demonstrated potentially pathogenic genetic variations in all 28 haplotypes from type I deficiency cases. While definitive assignment of pathogenicity benefits from population-scale frequency data (e.g., gnomAD), the complete co-segregation of these variants with the null phenotype in our cohort provides strong genetic evidence for their involvement. Compared with variants were detected in all haplotypes of type I deficiency samples, no *CD36* gene variants were detected in either haplotype in 7 type II deficiency samples, and even individuals carrying identical genotypes (e.g., I-02 and II-18) exhibited markedly different CD36 expression levels. These observations suggesting that other mechanisms-such as regulatory element alterations, aberrant splicing, or epigenetic modulation, or post-transcriptional regulation—may underlie type II deficiency, supporting the notion that type I and type II CD36 deficiency represent biologically distinct entities [23, 24].

The identification of a recurrent 19,971 bp deletion spanning exons 2–3 provides a strong candidate mechanism for CD36 type I deficiency. This deletion removes the canonical translation initiation site and the N-terminal coding region of *CD36*, which is predicted to abolish normal protein synthesis. In three type I cases, this structural variant was present on one haplotype, while the other haplotype carried a known loss-of-function variant. Notably, no additional rare or potentially pathogenic variants were detected on the deletion-bearing haplotypes, focused the candidacy of this deletion as the causative lesion on that haplotype.

This inference is further supported by prior molecular and transcriptional studies demonstrating that disruption of the 5’ region of *CD36*, including promoter-proximal exons, leads to profound loss of CD36 expression, thereby providing a strong mechanistic basis for a null phenotype [15, 26].

A key implication of this finding is its diagnostic invisibility to conventional genotyping methods [26, 27]. Standard exon-based PCR and Sanger sequencing interrogate only the presence and sequence of individual exons [28]. In heterozygous carriers of this deletion, exons 2 and 3 are readily amplified from the intact allele, producing apparently normal results and masking the deleted allele. As a result, compound heterozygotes may be misclassified as homozygotes for point mutations or remain genetically unresolved. This explains why a subset of CD36 type I deficiency cases has remained unexplained in prior studies and highlights a fundamental limitation of traditional exon-centered approaches.

From a clinical perspective, population screening revealed a 0.50% carrier frequency of this deletion. All heterozygous carriers showed normal CD36 expression, indicating absence of haploinsufficiency. However, compound heterozygosity, where this structural variant coexists with another potentially pathogenic allele, is predicted to result in a CD36-null phenotype. This has important implications for transfusion medicine, as cryptic carriers of this deletion may contribute to alloimmunization risk if not properly identified [29, 30].

## Conclusion

This study establishes the first full-length haplotype map of the *CD36* gene (~ 77 kb) through long-read sequencing of four overlapping amplicons. This approach enabled the discovery of a novel recurrent 19,971-bp deletion-insertion structural variant (c.1-15966_c.120 + 3887delinsCCAATGCTAAGGTTGA) spanning intron 1 to intron 3, which eliminates exons 2–3 and the translation initiation site. Importantly, by resolving phased haplotypes, we identified candidate genetic variations in all 14 cases of CD36 type I deficiency in this cohort. These findings provide substantial genetic evidence supporting a monogenic basis for this condition in the studied individuals. In contrast, no consistent genetic pattern was observed for type II deficiency, suggesting distinct etiological mechanisms. Together, our findings resolve a major diagnostic blind spot in *CD36* genotyping and highlight the necessity of long-read sequencing for accurate molecular diagnosis, population screening, and transfusion risk management.

## Supplementary Information

Supplementary Material 1.

## Data Availability

The raw sequencing data generated in this study have been deposited in the Genome Sequence Archive for Human (GSA-Human) in the National Genomics Data Center, China National Center for Bioinformation, Chinese Academy of Sciences (GSA-Human: HRA016850) that are publicly accessible at https://ngdc.cncb.ac.cn/gsa-human. All other relevant data supporting the findings of this study are available from the corresponding author upon reasonable request.

## Abbreviations

FNAIT: Fetal/Neonatal Alloimmune Thrombocytopenia
PTR: Platelet Transfusion Refractoriness
GPIV: Glycoprotein IV
RBCs: Red Blood Cells
SNV: Single Nucleotide Variant
UTRs: Untranslated Regions
PCR: Polymerase Chain Reaction
DNA: Deoxyribonucleicacid
EDTA: Ethylenediaminetetraacetic Acid
LR-PCR: Long-range PCR
PRP: Platelet-Rich Plasma
PBS: Phosphate Buffered Saline
BSA: Bovine Serum Albumin
MFI: Fluorescence Intensity
