# Supplementary material for: Full-length haplotype reconstruction of CD36 by long-read sequencing: uncovers a novel structural variant
Source: BMC Genomics. 2026 Feb 27;27:341. doi: 10.1186/s12864-026-12701-2 (PMC13049826; doi:10.1186/s12864-026-12701-2)
Supplement: Supplementary file 1 — Supplementary Material 1. [file 12864_2026_12701_MOESM1_ESM.docx]

**Supplementary Figure**


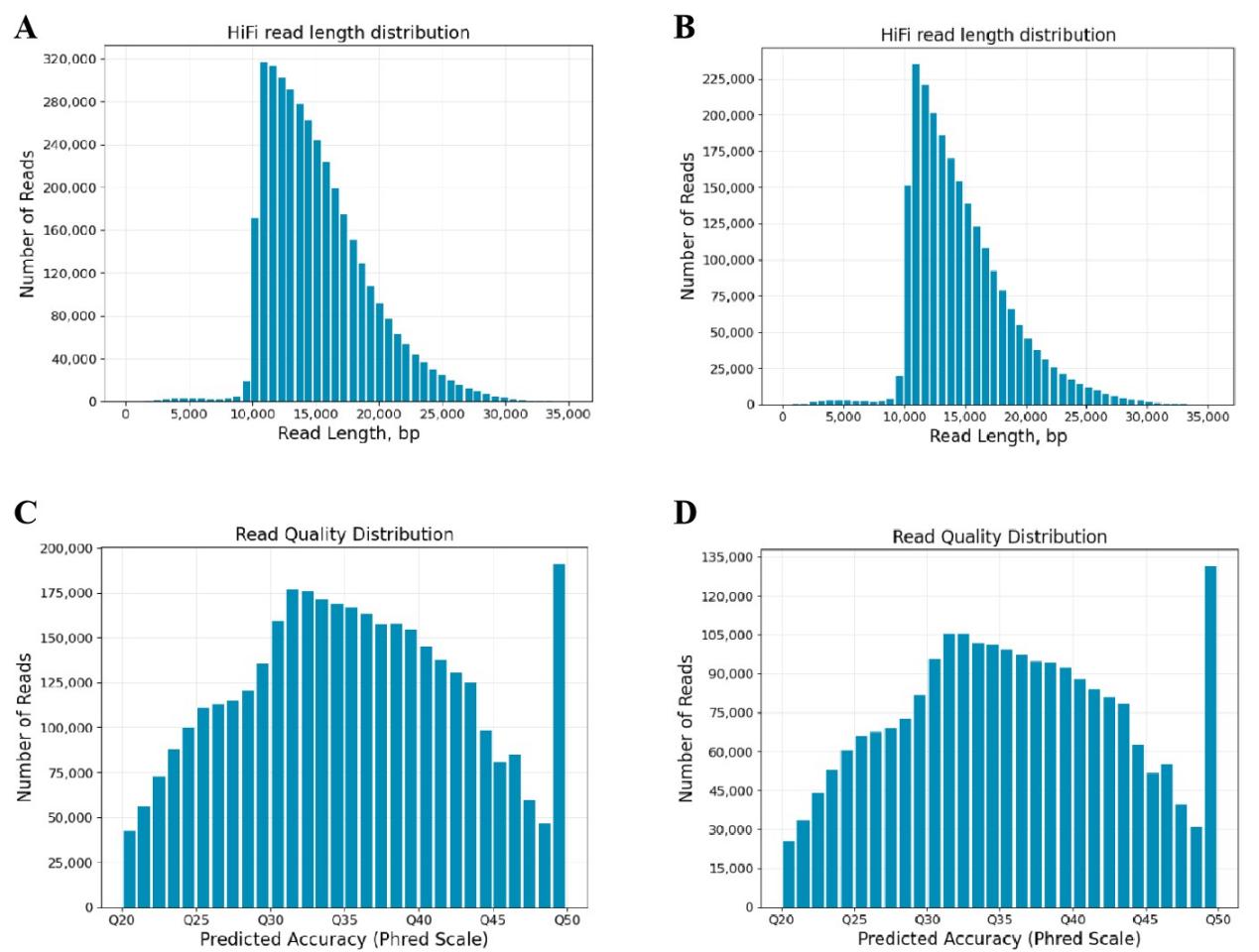


**Figure S1. HiFi read length and quality distributions from whole-genome sequencing.** (A, B) Read length distributions for samples I-13 and II-22, respectively. The majority of reads exceeded 10 kb. (C, D) Read quality score (Q-score) distributions for samples I-13 and II-22, respectively. Median Q-scores were Q35 for both samples.
